# Evolutionary Relationships and Divergence of *Filamin* Gene Family Involved in Development and Stress in Cotton (*Gossypium hirsutum* L.)

**DOI:** 10.3390/genes13122313

**Published:** 2022-12-08

**Authors:** Mingyang Wang, Lanxin Wu, Shouhong Zhu, Wei Chen, Jinbo Yao, Yan Li, Tengyu Li, Haihong Shang, Yongshan Zhang

**Affiliations:** 1 Zhengzhou Research Base, State Key Laboratory of Cotton Biology, Zhengzhou University, Zhengzhou 450001, China; 2 State Key Laboratory of Cotton Biology, Institute of Cotton Research, Chinese Academy of Agricultural Sciences, Anyang 455000, China

**Keywords:** *Filamin*, cotton, phylogenetic analysis, reproductive development, qRT-PCR, abiotic stress

## Abstract

Filamin protein is characterized by an N-terminal actin-binding domain that is followed by 24 Ig (immunoglobulin)-like repeats, which act as hubs for interactions with a variety of proteins. In humans, this family has been found to be involved in cancer cell invasion and metastasis and can be involved in a variety of growth signal transduction processes, but it is less studied in plants. Therefore, in this study, 54 *Filamin* gene family members from 23 plant species were investigated and divided into two subfamilies: FLMN and GEX2. Subcellular localization showed that most of the *Filamin* gene family members were located in the cell membrane. A total of 47 *Filamin* gene pairs were identified, most of which were whole-genome copies. Through the analyses of *cis*-acting elements, expression patterns and quantitative fluorescence, it was found that *GH_ A02G0519* and *GH_ D02G0539* are mainly expressed in the reproductive organs of upland cotton, and their interacting proteins are also related to the fertilization process, whereas *GH_A02G0216* and *GH_D02G0235* were related to stress. Thus, it is speculated that two genes of the GEX2 subfamily (*GH_A02G0519* and *GH_D02G0539*) may be involved in the reproductive development of cotton and may affect the fertilization process of cotton. This study provides a theoretical basis for the further study of the cotton *Filamin* gene family.

## 1. Introduction

Many proteins involved in the process of fertilization in flowering plants, such as *HAP2* (Hapless 2)/*GCS1* (generative cell specific 1) for gamete fusion [1,2,3,4,5], *KAR5* (karyogamy protein 5)/*GEX1*(gamete expressed 1) for nuclear fusion [6,7,8,9], *FUS1*/*GEX2* (gamete expression 2) for gamete adhesion and *GEX2*, contain special filamin-type immunoglobulin domains [10]. Filamin proteins, the first family of nonmuscle actin-binding proteins, are cytoplasmic proteins that cross-link cortical actin to form dynamic 3D structures. Each *Filamin* gene family member contains one or two filamin-type immunoglobulin (Ig) domains in animals [11,12,13]. The domain is constructed from a tandem repeat of 100 residue motifs rich in glycine and proline, followed by two β-sheets arranged into an Ig-like fold [14,15]. In the human body, the main role of the *Filamin* gene family is to combine with actins to form the cytoskeleton. It is a type of oncogenic protein involved in the invasion and metastasis of cancer cells and growth signal transduction processes [16,17]. In plants, a member of the *Filamin* gene family that contains only one Filamin domain has been reported to be the basis of post-transcriptional, auxin-mediated gene expression in *Arabidopsis* [18], and is a key defense regulator against *Arabidopsis* pathogens [19]. A family member with two Filamin domains, named *GEX2*, has been extensively studied in recent years and is mainly involved in the process of double fertilization in plants. In 2005, *AtGEX2* (*At5g49150*), belonging to the *Filamin* gene family, was found to be active in sperm cells but not in other vegetative cells, and *GEX2* is required for gametophyte attachment in *Arabidopsis* [20,21,22,23]. In maize, *GEX2* is also localized to sperm membranes and has been found to be involved in gametophyte fusion [24,25,26]. In *Chlamydomonas*, *GEX2* is also a plant sperm protein with an adhesive function, which can bind to *HAP2* and play a role in gametophyte recognition and adhesion [27,28]. The *GEX2* subfamily with an Ig-like domain can play a role in the gametophyte. According to the functions of these genes in *Arabidopsis*, maize and *chlamydomonas*, it is speculated that *Filamin* gene family members in other plants may also participate in the reproductive development process of plants and play a role in sperm recognition and adhesion.

Cotton is one of the most important cash crops in the world, which provides us with important natural fibers and oil seeds. Research on cotton reproduction and development is of great significance for cotton breeding. At present, the *Filamin* gene family has only been found to be involved in gametophyte fusion in *Arabidopsis*, maize and *chlamydomonas*, but there has been no systematic analysis of the whole family with regard to plants. In cotton, this family has never been systematically studied. Whether this gene family has the same function in cotton is a question worth exploring. Therefore, a series of analyses and research have been carried out on the cotton *Filamin* gene family. In this study, 54 genes were identified in 23 species, which were divided into two subfamilies, FLMN and GEX2. Both subfamilies contained 1–2 filamin domains, and four genes related to reproductive development and abiotic stress were identified via a *cis*-element analysis and qRT-qPCR analysis. This research further clarifies the role of the *Filamin* gene family in plants, and provides candidate genes for further functional studies in addition to establishing a theoretical basis.

## 2. Material and Methods

### 2.1. Databases and Whole-Genome Identification of Filamin Gene Family Members in Plants

The genome databases of four cotton species were downloaded from CottonFGD (http://cottonfgd.org, accessed on 9 May 2022) [29], including *Gossypium arboreum* (ZJU, version 1.0), *Gossypium raimondii* (ZJU, version 2.0), *G. hirsutum* (ZJU, version 1.0) and *Gossypium barbadense* (ZJU, version 1.0). Additionally, all 19 species, including *Arabidopsis thaliana* (TAIR, version 10), *Zea mays* (Ensembl-18_2010_01), *Amborella trichopoda* (version 1.0), *Glycine max* (version 1.0), *Oryza sativa* (version 7.0), *Sorghum bicolor* (version 3.1.1), *Selaginella moellendorffii* (version 1.0), *Theobroma cacao* (version 2.1), *Vitis vinifera* (version 2.1), *Zostera marina* (version 2.1)*, Chlamydomonas* (version 2.1)*, Physcomitrium patens* (version 2.1)*, Panicum miliaceum* (version 2.1)*, Setaria italic* (version 2.0), *Aquilegia viridiflora Pall* (version 2.1)*, Ananas comosus Populus* L. (version 3.0)*, G. max* (version 3.1) and *Brachypodium distachyon* (version 2.1), were acquired from Phytozome (https://phytozome-next.jgi.doe.gov/ (accessed on 10 May 2022)) [30].

Twenty-three species of plants were blasted with known *Filamin* gene family members in animals by using TBtools [31]. The obtained sequences were batch searched by using the Pfam (http://pfam.xfam.org/, accessed on 11 May 2022) [32] batch searches with the default setting of the threshold option, the Conservative Domain Database (CDD) batch search (https://www.ncbi.nlm.nih.gov/cdd/, accessed on 11 May 2022) [33] and the SMART database batch search (http://smart.embl-heidelberg.de/, accessed on 11 May 2022) [34] in order to determine the protein sequences of candidate genes. The physicochemical properties of each protein sequence were obtained by using ExPASY (https://web.expasy.org/compute_pi/, accessed on 11 May 2022) [35], and the subcellular localization of each candidate sequence was predicted by using CELLO (http://cello.life.nctu.edu.tw/, accessed on 12 May 2022) [36,37] to determine the specific expression position of this protein in cells.

### 2.2. Phylogenetic Tree Construction and Sequence Analysis of Filamin Gene Family Members

The Clustalw option in the MEGA 7.0 software [38] was used to align peptide sequences of filamin proteins of 23 species and constructs an unrooted phylogenetic tree. In order to clearly understand the evolutionary relationship of the *Filamin* gene family among the four cotton species, ML trees were constructed for the four cotton species separately. The maximum-likelihood method was used and 1000 replications were bootstrapped.

### 2.3. Chromosomal Distribution, Gene Duplication and Synteny Analysis of the Filamin Gene Family Members for Four Cotton Species

The location of the cotton *Filamin* gene family members on the chromosome can be obtained using the GFF3 file and, subsequently, visualized by using TBtools. A collinearity analysis was performed on four cotton species to obtain homologous and par-homologous gene pairs, and collinearity maps were visualized by using the Advanced Circos function of the TBtools software. *G. raimondii* and two representative species (*V. vinifera* and *T. cacao* were subjected to a homolinear analysis via the TBtools’ One Step MCScanX and Text Merge for MCScanX.

### 2.4. Calculation of Ka/Ks of Filamin Gene Family Members in Three Cotton Species

Orthologous gene pairs of the *Filamin* gene family members were identified in *G. raimondii*, *G. arboretum* and *G. hirsutum* by using TBtools. The Ka/Ks ratio of all the homologous gene pairs was calculated using the Ka and Ks values of the obtained orthologous gene pairs via the Kaks_Calculator 2.0 program [39,40].

### 2.5. Gene Structure, Conserved Motif and Gene Ontology Analysis of Filamin Gene Family

The position information of gene exons and introns was found through the GFF3 file and visualized by GSDS (http://gsds.gao-lab.org/, accessed on 14 May 2022) [41,42]. The Motif Elicitation (MEME) website (https://meme-suite.org/meme/tools/meme, accessed on 14 May 2022) [43] was used to predict ten conserved motifs of four cotton species, where the *p*-value of each motif on each protein was lower than 1 × 10^−5^. The conserved domains were predicted by using the NCBI’s Batch Web CD-Search Tool (https://www.ncbi.nlm.nih.gov/Structure/bwrpsb/bwrpsb.cgi, accessed on 14 May 2022) [44,45], where the e-value of each motif on each protein was lower than 0.01, and finally, they were visualized and merged. A GO analysis was performed through the OmicShare company’s online program (https://www.omicshare.com/tools/Home/Soft/gogseasenior, accessed on 14 May 2022). The signal peptide and transmembrane domain of each filamin protein were predicted by using SignalP-5.0 (https://services.healthtech.dtu.dk/service.php?SignalP-5.0, accessed on 14 May 2022) [46] and TMHMM-2.0 (https://services.healthtech.dtu.dk/service.php?TMHMM-2.0, accessed on 14 May 2022) [47], and were then visualized.

### 2.6. 3D Structure of Filamin Protein in G. hirsutum

In order to understand the structure of filamin proteins more clearly, the homology modeling of the tertiary structure of filamin proteins was carried out by using the SWISS-MODEL website (https://swissmodel.expasy.org/, accessed on 16 May 2022) [48], and the reliability of the tertiary structure was evaluated according to the sequence similarity and model score.

### 2.7. Promoter Region Cis-Element Analysis and Expression Profile Analysis of Different Filamin Gene Family Members

The upstream 2000 bp sequence of the *Filamin* gene family member promoter of upland cotton was extracted via TBtools. The *cis*-elements in these promoter regions were predicted via PlantCARE (http://bioinformatics.psb.ugent.be/webtools/plantcare/html/, accessed on 18 May 2022) [49,50,51], and were screened and classified. They mainly included three types of *cis*-acting elements (phytohormones, plant growth and abiotic stress). To analyze the expression data of *Filamin* gene family members in upland cotton under different tissues and stresses, genome-wide RNA-seq data of each gene were downloaded from the Cotton Omics Database [52] (http://cotton.zju.edu.cn/, accessed on 20 May 2022). The heatmap charts were drawn according to gene expression values (FPKM) by using TBtools.

### 2.8. RNA-Seq and Quantitative RT-PCR (qRT-PCR) for Filamin Gene Family Members in G. hirsutum

A qRT-PCR was used to analyze the expression of six *GhFilamin* genes in various tissues and under different abiotic stress conditions. Different tissues including roots, stems, leaves, sepals, bracts, petals and anthers (flower bud size <3 mm, 4–5 mm, 5–8 mm and >8 mm; the anther stages were compared to those described by [53]), pollen, ovules (0DPA, 1DPA, 3DPA, 5DPA, 10DPA and 20DPA) and fibers (10DPA, 20DPA and 25DPA)) of upland cotton were obtained from Zhongmiansuo 100. After field sampling, they were put into liquid nitrogen to be cold shocked, and then were frozen at −80 °C and put on standby. In the stress treatments, seedlings were exposed to abiotic stresses of heat (37 °C for 1, 3, 6, 12, 24 and 48 h), cold (4 °C for 1, 3, 6, 12, 24 and 48 h), salt (200 mM NaCl for 1, 3, 6, 12, 24 and 48 h) and 20% polyethylene glycol (PEG 6000 for 1, 3, 6, 12, 24 and 48 h) [54]. Immediately following sampling, samples were frozen in liquid nitrogen and stored at −80 °C. The total RNA was isolated with the RN38-EASYspin-Plus Plant RNA Kit (Aidlab Co., Ltd., Beijing, China) according to the manufacturer’s instructions, and the PrimeScriptTM RT Reagent Kit with a gDNA Eraser (Vazyme Biotech Co., Ltd., Nanjing, China) was used to reverse transcribe the RNA. qPCR primers were designed on the qPrimerDB-qPCR Primer Database website (https://biodb.swu.edu.cn/qprimerdb/manual, accessed on 20 May 2022) and *Ghactin7* was used for the housekeeping gene (Table S5). Finally, a 7500 Real-Time PCR System (Bio-Rad, Hercules, CA, USA) was used to perform the PCR with the SYBR-Premix Ex Taq Kit from Takara (Takara Biomedical Technology Co., Ltd., Beijing, China). qPCR cycles were performed as follows: 95 °C for 5 min, followed by 40 cycles of amplification (95 °C for 10 s and 60 °C for 40 s). The 2^−ΔΔCt^ method was used to determine the relative gene expression after the experiments were repeated three times [55].

### 2.9. Protein Interaction Network Analysis of Filamin Proteins

Construction of a protein–protein interaction (PPI) network: A PPI network was constructed for the whole family and filamin proteins of *A. thaliana* through the Search Tool for the Retrieval of Interacting Genes (STRING) Database (https://cn.string-db.org/, accessed on 20 May 2022) [56], with a confidence parameter set to a threshold of 0.15.

## 3. Results and Discussion

### 3.1. Identification, Physicochemical Property Analysis and Subcellular Localization Prediction of Filamin Proteins

By conducting a blast search in TBtools against known animal filamin protein sequences [31], the filamin proteins of 23 plants were identified (Figure S1 and Table S1). Following that, SMART and Pfam were used to verify whether each protein sequence contains a filamin domain (PF00630) [32,34]. A total of 54 *Filamin* gene family members were identified from 23 plants, of which 18 were identified to be from 4 cotton species, including 3 from *G. arboretum* (Ga), 3 from *G. raimondii* (Gr), 6 from *G. hirsutum* (Gh) and 6 from *G. barbadense* (Gb). The number of *Filamin* gene family members in tetraploid cotton (island cotton and upland cotton) is exactly twice that of diploid cotton (*G. raimondii* and *G. arboretum*), which proves sufficiently that the allotetraploid of cotton is formed by doubling the chromosomes after hybridization between the A-genome diploid and D-genome diploid. In contrast, many species contain one or two genes. Among them, *Z. marina*, *V. vinifera, T. cacao, A.trichopoda, Chlamydomonas* and *B. distachyon* all contain one *Filamin* gene family member, whereas *S. lycopersicum*, *S. bicolor*, *P. patens*, *P. miliaceum*, *O. sativa*, *S. italica*, *M. balbisiana Colla*, *A. viridiflora*, *A. comosus* and *A.thaliana* all contain two *Filamin* gene family members. Additionally, *Populus L* and *S.tamariscina* contain three *Filamin* gene family members and contain four *Filamin* gene family members, respectively. According to the identification results, it can be seen that the number of genes of upland cotton and island cotton is the largest, which is, basically, 2–6 times that of other species, showing that two allotetraploid cottons had expanded and replicated in the evolutionary process. This fully proves that over the long evolution process of *Filamin* gene family members, many species have experienced the problems of gene loss and evolutionary imbalance.

Subsequently, the physicochemical properties of all genes were analyzed by using ExPASy [35]: the molecular weight ranged from 70.329kDa (evm_27modelAmTr_v1.0_scaffold00013239) to 209.589kDa (Solyc09g092250); the proteome isoelectric point was from 4.57 (407438) to 10.67 (GH_A02G0216); and all the Gravy was negative value, indicating that filamin proteins were hydrophilic proteins. Results from the predicted subcellular localization showed that the filamin proteins are mainly distributed on the outer membrane of the cell and that some of the proteins are also found in the extracellular and periplasmic. In *G. raimondii*, *Gorai005G061500*, belonging to the GEX2 subfamily, is found only in the outer membrane, whereas two genes belonging to the FLMN subfamily are found both in the outer membrane and outside the cell. In upland cotton, however, the situation is somewhat different. *GH_A02G0216*, which belongs to the FLMN subfamily, is found only in the extracellular membrane, but not outside the cell. In summary, the GEX2 subfamily proteins were localized only on the extracellular membrane in 23 plant species, whereas the FLMN subfamily proteins were localized not only on the extracellular membrane, but also in the periplasm (Table S1).

### 3.2. Sequence and Evolutionary Analysis of Filamin Gene Family Members in 23 Plant Species

**Figure 1:**
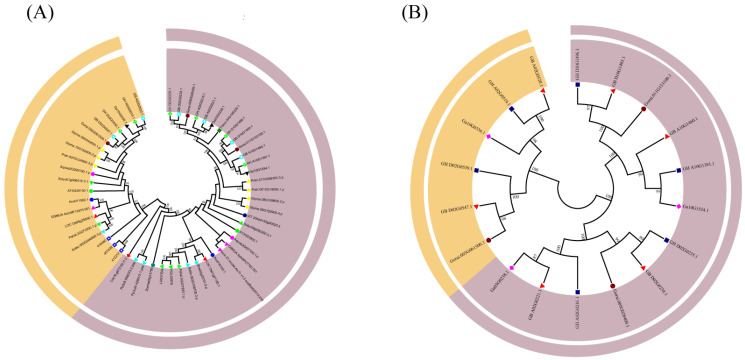
Two unrooted phylogenetic trees were constructed via the MEGA7 maximum-likelihood method. The percentage of replicate trees in which the associated taxa clustered together in the bootstrap test (1000 replicates) is shown next to the branches. All positions with less than 90% site coverage were eliminated. (**A**) Phylogenetic relationship of the 54 identified *Filamin* gene family members from *Gossypium raimondii* and 19 other plant species. (**B**) A phylogenetic tree of *Filamin* gene family members in four cotton species.

*Filamin* gene family members in plants were obtained by blast of known animal *Filamin* gene family members. The sequence analysis and phylogenetic tree construction of 23 species were performed by using MEGA7 (Figure 1A) [38]. They can be divided into two main groups based on clustering and previous studies: the GEX2 subfamily and FLMN subfamily. Among them, the GEX2 subfamily has been studied in *A. thaliana* and maize in recent years, and thus, the name of this subfamily is reserved. The evolutionary tree showed that the number of GEX2 subfamilies was smaller than that of FLMN subfamilies, and many species did not contain GEX2, indicating that some of the *Filamin* gene family members of this species were lost through evolution, and only the members of the FLMN subfamily were retained. The distribution of *Filamin* gene family members in each genome is not uniform. These results indicate that *Filamin* gene family members existed in the ancestral genome before the separation of dicots and monocots, and then were distributed in the genomes of monocots and dicots after different differentiation times. In the GEX2 subgroup, the copy number was generally between one and two, with only *S.tamariscina* being the highest as it contains three copies, but there are many species that do not contain the GEX2 gene. However, in the FLMN subgroup, almost every species contained one or two *Filamin* gene family members; only *S.tamariscina* did not, indicating that *S.tamariscinaFilamin* gene family members were assigned to the GEX2 subgroup in the evolutionary process.

In order to further investigate the relationship between *Filamin* gene family members in diploid cotton and tetraploid cotton, the sequence analysis and phylogenetic tree construction were carried out separately for four cotton species (Figure 1B). It is clear from the evolutionary tree that there is twice as many allotetraploid cotton as diploid cotton in both subgroups, and that for each diploid cotton species, there are two corresponding allotetraploid cotton species. It was proved once again that the allotetraploid cotton species evolved from a hybrid of the diploid cotton species and that during the evolution of the cotton *Filamin* gene family members, they underwent stable selection and no gene loss occurred.

### 3.3. Chromosome Distribution of Filamin Genes for Four Cotton Species

The chromosome distribution map further clarifies the characteristics of evolution and replication of *Filamin* gene family members. The results show that 18 *Filamin* gene family members are located on 12 different chromosomes (Figure S2). In *G. arboretum*, a total of three genes are located on two chromosomes (Chr03 and Chr10), but in *G. raimondii*, the three *Filamin* gene family members are on two chromosomes, Gr-Chr05 and Gr-Chr11. In *G. hirsutum*, there were two *Filamin* gene family members on chromosomes Gh-A02 and Gh-D02, and one gene on chromosomes Gh-A10 and Gh-D10. Similarly, *G. barbadense* also contains two *Filamin* gene family members on Gb-A02 and Gb-D02, and one gene on Gb-A10 and Gb-D10. According to the results, there are no *Filamin* gene family members located on the scaffold, indicating that *Filamin* gene family members are relatively mature in regard to the process of evolution. The distribution of *G. raimondii* and *G. arboretum* on different chromosomes may be due to the random distribution of genes during evolution. However, the distribution of *G. hirsutum* and *G. barbadense* on the chromosome is relatively uniform, the evolution is relatively mature, and there is no gene loss and chromosome recombination event. Interestingly, the copy numbers of the At and Dt subgroups of both allotetraploid cotton species were identical, at about 1:1. Moreover, their numbers and chromosome distributions were also completely consistent, which further confirmed that *Filamin* gene family members were relatively conserved in the evolution of the two cotton species. In addition, the number of genes in subgroup At and subgroup Dt of the two allotetraploid cotton species was consistent with the number of genes in diploid cotton, and the distribution position was roughly the same, indicating that allotetraploid cotton was crossed from two diploid cotton species.

### 3.4. Collinearity Analysis and Ka/Ks Analysis of Filamin Gene Family Members

**Figure 2:**
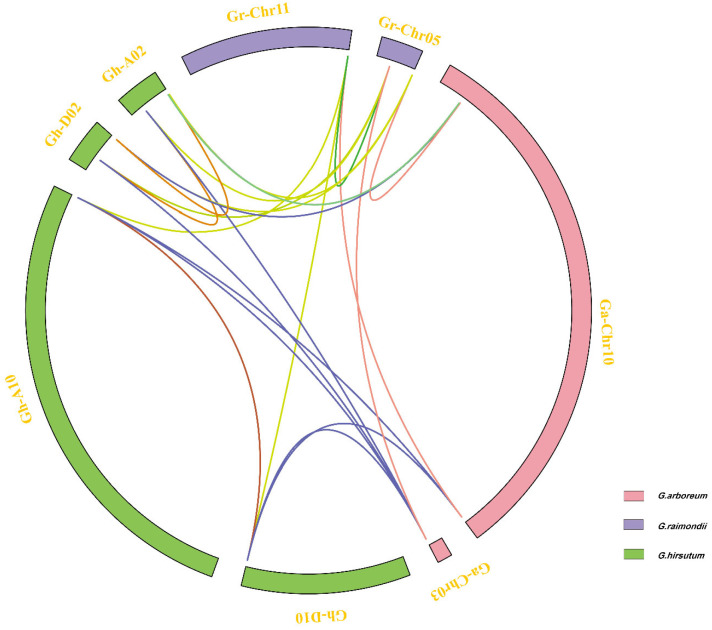
The collinearity relationships of *Filamin* gene family members of three cotton species. The chromosomes of *G. arboreum*, *G. raimondii* and *G. hirsutum* are shown with pink, blue and green colors, respectively.

**Figure 3:**
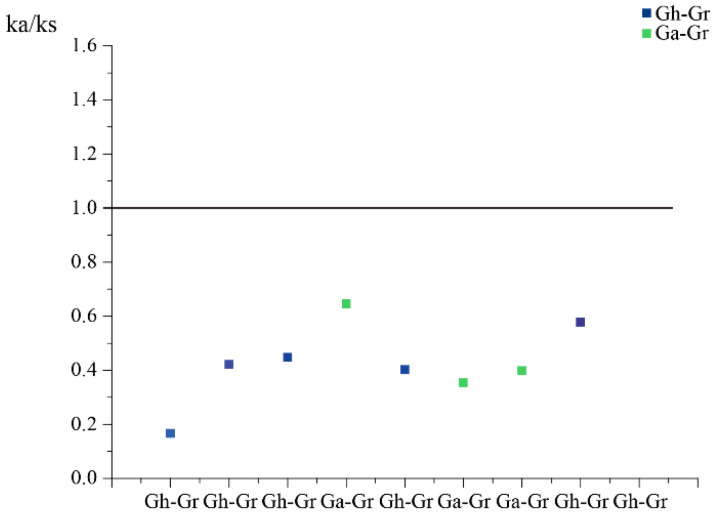
Analysis of Ka and KS ratio of three cotton species. The blue square stands for Gh-Gr; the green square stands for Ga-Gr.

A total of 47 orthologous/paralogous gene pairs were found via a collinearity analysis in three cotton species (*G. raimondii*, *G. arboretum* and *G. hirsutum*), and almost all collinearity pairs are whole-genome duplications (WGDs) (Figure 2, Table S2). These results indicate that the amplification of the *Filamin* gene family members in cotton mainly depends on whole-genome replication. In addition, there are two pairs of tandem repeats in upland cotton. It can be inferred that in the evolution process of *Filamin* gene family members in cotton, the homologous genes are usually completed by whole-genome replication and tandem replication, and the whole-genome replication is the main driving force. In the process of cotton gene doubling, a large number of chromosome rearrangements, inversions, translocations and gene rank segment losses are usually experienced, which promote the evolution of cotton *Filamin* gene family members. By choosing two more representative species (*T. cacao* and *V. vinifera*) to compare with *G. raimondii* through a linear analysis (Figure S3), it can be clearly seen that *G. raimondii* and *T. cacao* only have one syntenic gene pair, which does not exist between *G. raimondii* and *V. vinifera*. According to the Gr-Ga and Gr-Gh collinearity of the relationship between the computing Ka/Ks ratio, it can be clearly seen that most genes have undergone purification selection (Figure 3), and there is only one gene for positive choice. This family is highly conservative during cotton evolution, and this strong purification selection also increases the redundancy of cotton Filamin gene family functions.

### 3.5. Gene Structure and Conserved Domain Analysis of Filamin Gene Family Members in Cotton

**Figure 4:**
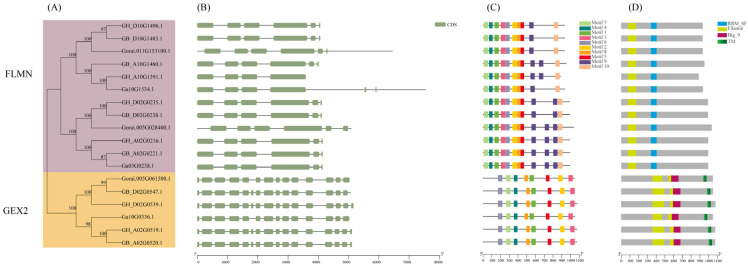
The gene structure, conserved motifs and domains in *Filamin* gene family members of four cotton species. (**A**) The ML phylogenetic tree was constructed based on the full-length sequences of four cotton species *Filamin* gene family members by using MEGA 7.0 software. Different subfamilies are indicated by different colors. (**B**) Exon–intron structure of four cotton species *Filamin* gene family members. Light green for exons; black lines for introns. (**C**) Conserved motif of four cotton species. Different colored squares represent different motifs, named motif1–motif10. (**D**) The conserved domains in four cotton species *Filamin* gene family members.

The coding regions of genes are usually composed of different numbers of exons and introns. The *Filamin* gene family members in cotton are mainly composed of several introns and exons (Figure 4B). In the GEX2 subfamily, almost every gene contains 14–16 exons and 13–15 introns. In the FLMN subgroup, the number of exons of this gene is very small, basically only containing 4–6 exons and 3–5 introns. The number of exons and introns of members of the same subgroup is basically the same, indicating that each subfamily is highly conserved in evolution and relatively mature. The gene structure of the members of different subfamilies is distinct, and the genes with a similar gene structure are clustered together, which also confirms the close relationship between the gene genetic structure, evolution and phylogeny.

**Figure 5:**
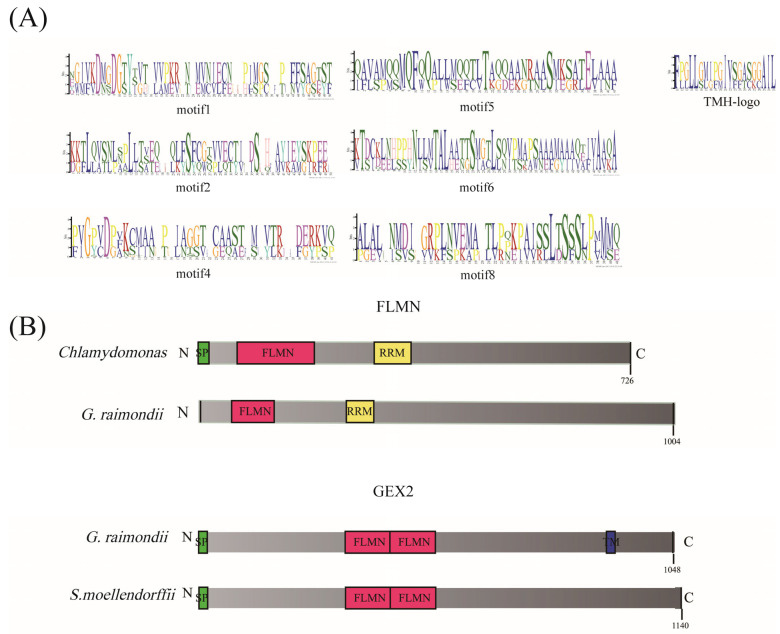
(**A**) Sequence logos of conserved motifs (Motif1, Motif2, Motif4, Motif5, Motif6 and TMH-logo); (**B**) domain arrangement model of filamin proteins’ domain in space. SP stands for signal peptide, TM stands for transmembrane region.

The conserved motifs of *Filamin* gene family members were predicted by using MEME, and a total of 10 motifs were predicted and named Motif1–Motif10 (Figure 4C and Figure S4). Among them, there is a transmembrane structure in Motif3, which may be a functional site (Figure 5). In the FLMN subfamily, each gene of cotton contains these 10 conserved motifs, whereas in the GEX2 subfamily, Motif9 and Motif10 are not contained, and only 8 conserved motifs are contained. This indicates that the *Filamin* gene family members have been differentiated in the process of evolution, and these changes have also led to changes in gene function. For example, the GEX2 family with two conserved motifs plays a role in gametophyte recognition, attachment and membrane fusion in flowering plants during fertilization, whereas the function of the FLMN subfamily needs to be studied. In the FLMN subgroup, it can be found that there are 2–3 repeats of Motif9 in some genes, which may be a new function of this motif in the evolutionary process. The other motifs were basically identical in the two subgroups, especially in the GEX2 subgroup, where each motif was identical without any duplication. This fully indicates that *Filamin* gene family members are highly conserved in cotton evolution. The different distribution of motifs also indicates the diversity of gene functions.

Through the prediction and visualization of the conserved domains in the cotton sequence, it can be seen that there are four typically conserved domains: filamin, RRM-SF, Big-9 and TM (Figure 4D). There is no doubt that the filamin domain is the domain that is unique to the *Filamin* gene family. RRM-SF is an RNA recognition site, and this domain exists, both in cotton and in other species, only in the FLMN subfamily but not in GEX2. It may be involved in the regulation of alternative splicing, the composition of small ribosomal nucleoproteins and the regulation of RNA stability and translation, but its specific function in the *Filamin* gene family remains to be investigated. There are also two conserved domains in the cotton GEX2 subfamily, BIG-9 and TM. TM is a common transmembrane structure, usually located at the C-terminus of the protein sequence, which may further confirm the function of GEX2 in gametophyte recognition, attachment and fusion. Big-9 is a bacterial Ig domain that is widespread in the GEX2 subfamily, but absent from only two genes in *Selaginella.* In addition to these domains, four genes, *Pahal5G437200*, *KQL03978*, *KQL03977* and *Aco016105,* contain ZNF-C3H3, also known as Nab2 ZNF5-7 [57], an unusual zinc finger family; Nab2 is a conserved polyadenosine RNA-binding zinc finger protein required for mRNA export and polyadenylation regulation and is linked to mRNP after splicing, and during or immediately after polyadenylation, whereas the three ZNF5-7 family gene members form a coherent structural unit and chemical shift perturbations identify residues on each finger that interact with RNA.

### 3.6. Protein 3D Structure Prediction of Filamin Proteins

**Figure 6:**
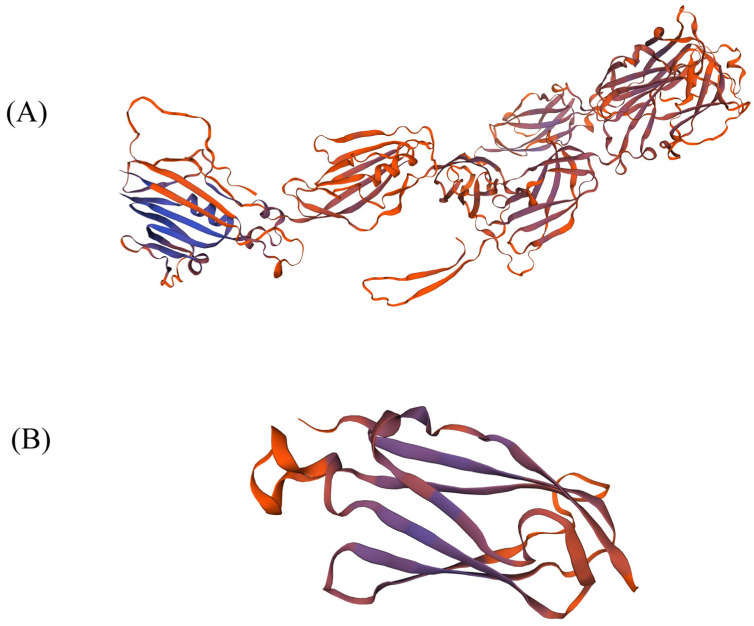
Tertiary structure of filamin proteins in upland cotton. (**A**) Tertiary structure of GEX2 subgroup protein in upland cotton. (**B**) Tertiary structure of FLMN subgroup protein in upland cotton.

The difference in protein tertiary conformation leads to the difference in protein function, and the protein tertiary structures of the GEX2 and FLMN subfamilies are very different. The PDB file of the *Arabidopsis* filamin proteins, which is closest to *GH-D02G0539* and *GH-D02G0235*, was downloaded from the AlphaFold website (https://alphafold.ebi.ac.uk/, accessed on 5 June 2022) [58]. The predicted structure of the GEX2 protein was judged according to its GMQE score, QMEAN and sequence similarity. The GMQE score was about 0.4, the QMEAN value was −1.9 and the sequence similarity was 57.7%. The model quality was reliable (Figure 6A). It is clear that the GEX2 subfamily of proteins is a polymer composed of α-helice and β-sheet. The tertiary structure of FLMN subfamily proteins is relatively simple, consisting of only a few α-helice and β-sheet that form a monomer. The GMQE score of the model was 0.1, the QMEAN value was 0.58 and the sequence similarity was 27.66%. The quality of the model was available for reference (Figure 6B). The differences in the protein tertiary structures may also contribute to the functional differences between the two subfamilies.

### 3.7. Analysis of Promoter Cis-Elements of Filamin Gene Family Members in Upland Cotton

**Figure 7:**
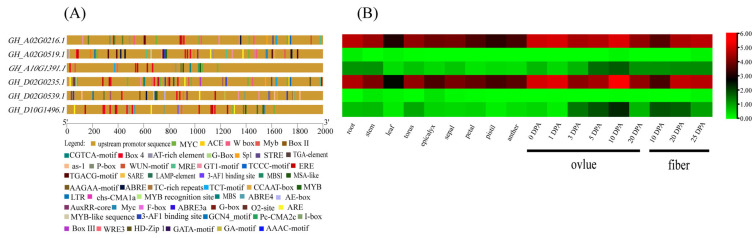
Analysis of *Filamin* gene family member promoter and its expression pattern in different tissues. (**A**) *Cis*-elements in promoters of *Filamin* gene family members. (**B**) Expression pattern of *Filamin* gene family members in different tissues.

There are 239 specific *cis*-acting elements in the six upland cotton *Filamin* gene family members from PlantCARE (Figure 7A, Table S3); the most important ones are growth and development elements, of which there are 103 in total, accounting for 43%. Light-responsive elements are the most abundant of these components, with a total of 90 including ACE, Box4, G-Box, Sp1, MRE, the GT1-motif, the TCCC-motif, the TCT-motif, chs-CMA1a, the 3-AF1 binding site, LTR, etc. This fully indicates that *Filamin* gene family members of upland cotton are regulated by light. The remaining 13 reaction elements include zein metabolism regulation (O2-site) and endosperm expression (GCN4_motif), which may be involved in the expression of endosperm and the metabolism of zein. There are 86 reaction elements of hormones, which account for about 35% of the total. Among them, the most common response elements are abscisic acid-responsiveness elements (AAGAA-motif, ABRE and ABRE4), totaling about 26; followed by MeJA-responsiveness elements (CGTCA-motif and TGACG-motif), totaling about 24; and the least common response element is gibberellin-responsive elements (P-box), totaling only one. It is speculated that this gene may be involved in upland cotton in the reproductive development and the senescence process. The smallest number of abiotic stress response elements was only 49, and the largest number of dehydration response elements (MYC, Myb and Myc) was about 27, indicating that *Filamin* gene family members were more susceptible to water loss stress in cotton. There are only four drought-inducibility response elements (MBS), two defense- and stress-responsive elements (TC-rich repeats,) and one low-temperature-responsive element (LTR), which may indicate that *Filamin* gene family members of upland cotton are rarely threatened by these abiotic stresses or their expression is not greatly affected. There is also a special transcription factor HD-ZIP1 that plays an important role in responding to the abiotic stress of plants and indirectly affects the growth and development of plants to different degrees, leading to growth retardation, senescence, production reduction and even the death of plants.

### 3.8. Expression Pattern Analysis of Filamin Gene Family Members in Upland Cotton under Different Tissues and Abiotic Stresses

**Figure 8:**
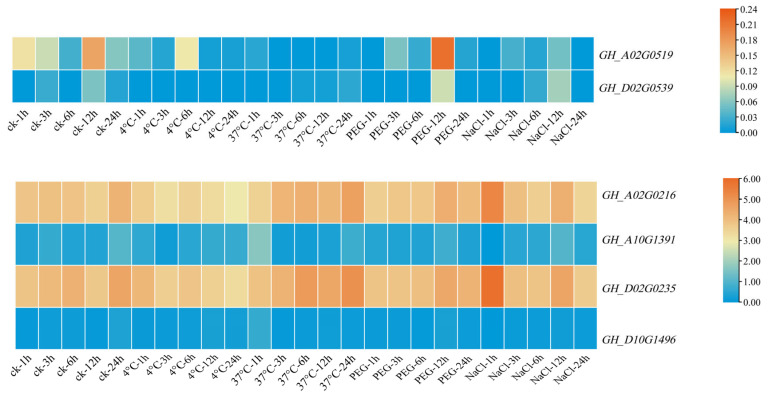
Expression pattern of *Filamin* gene family members under abiotic stresses of *G. hirsutum*.

The expression data of six *GhFilamin* gene family members in upland cotton under different tissues and different stress conditions were downloaded from the public database of Zhejiang University and were visualized (Figure 7B, Table S4, Figure 8).

These six genes are basically expressed in the development of reproductive organs (petals and ovules) and fibers, indicating that these genes may be related to the reproductive development of upland cotton. In the GEX2 subfamily, *GH_A02G0519* was highly expressed in the roots, leaves, and 10DPA and 25DPA fibers, and its homologous gene *GH_D02G0539* was specifically expressed in the stems, leaves, sepals, epicalyxes and 25DPA fibers, and was also expressed in petals, 1DPA ovules, 10DPA fibers and 20DPA fibers, but not in other tissues. Among the four genes in the FLMN subfamily, *GH_A10G1391* and *GH_D10G1496* were mainly expressed in the ovules and fibers, and *GH_A0G1391* was slightly higher in the 5DPA ovule and 10DPA ovule. *GH_D10G496* was higher in the 5DPA ovule, 10DPA ovule, and 10DPA and 20DPA fibers. The situation of *GH_A02G0216* and *GH_D02G0235* is similar, and the specific expression of *GH_A02G0216* and *GH_D02G0235* was found in the development of ovules and fibers at various stages. The expression patterns of these six genes were verified via qRT-PCR at a later stage to further explore the expression patterns of these six genes.

*Filamin* gene family members are also affected by many abiotic stresses in the development process; through the analysis of RNA-seq data, it can be obviously found that the expression levels of these genes are downregulated to different degrees when subjected to abiotic stress. In subgroup GEX2, *GH_A02G0519* was downregulated to different degrees under various stresses, but the change was not obvious. What was more special was that, in addition to other stresses, the expression of this gene showed a significant upward trend after a 4 °C treatment for 6 h and a PEG treatment for 12 h compared with the control. *GH_D02G0539* and *GH_A02G0519* had the same change trend when subjected to abiotic stress, which showed an overall downregulation trend, and showed an upregulation trend after 6 h of a low-temperature treatment and 12 h of a PEG treatment. In the other subgroup, the expression levels of *GH_A02G0216* and *GH_D02G0235* decreased significantly with the low-temperature treatment, whereas the expression levels of *GH_D02G0235* increased continuously with the high-temperature treatment. The expression levels of *GH_A02G0216* and *GH_D02G0235* increased significantly at 1 h after a NaCl treatment, and then decreased at 24 h. However, the changes were small after the PEG treatment, indicating that these two genes changed significantly due to temperature stress, which may be related to the *cis*-element LTR. After low-temperature treatment, the expression of *GH_A10G1391* basically increased, whereas under a high-temperature treatment, except for the 1 h treatment, the expression of cotton had an obvious upward trend, and the other treatment time showed down-regulated. The expression of PEG and NaCl decreased after treatment. The change trend of *GH_D10G1496* was different from that of *GH_A10G1391*, and the expression of *GH_D10G1496* was upregulated after PEG treatment. A qRT-PCR analysis of these six genes was performed to further confirm the accuracy of RNA-seq data and provide data support for subsequent experiments.

### 3.9. qRT-PCR Analysis of Filamin Gene Family Members in Upland Cotton under Different Tissues and Abiotic Stresses

**Figure 9:**
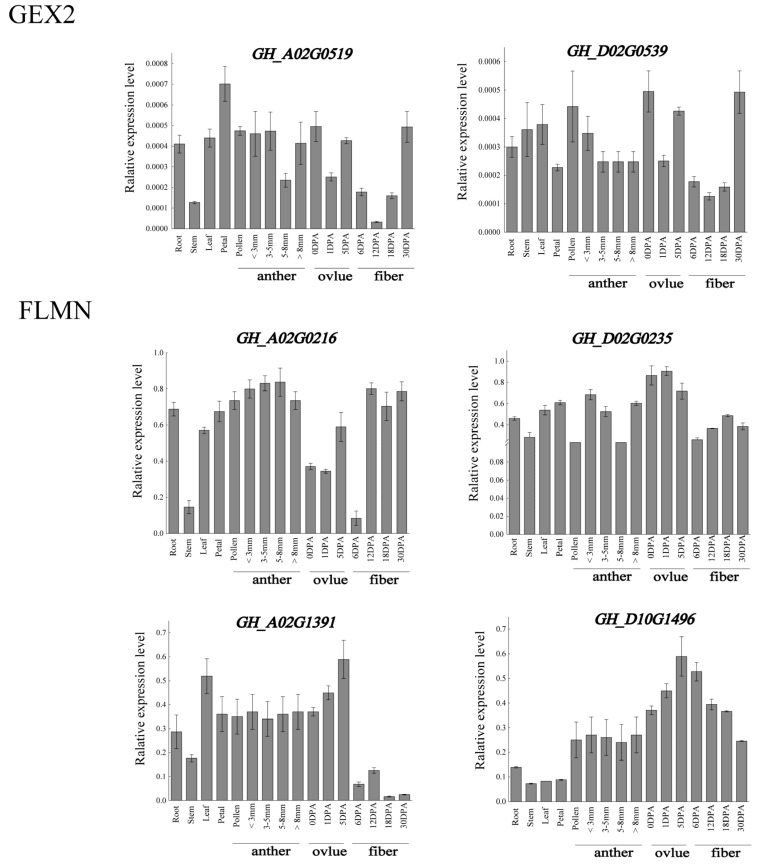
The expression of *Filamin* gene family members in different tissues of *G. hirsutum*.

In order to verify the expression of *Filamin* gene family members in upland cotton under various tissues and abiotic stress, a qRT-qPCR analysis of these six genes was carried out. By analyzing the expression levels of six upland cotton genes in various tissues, it was obvious that the expression levels of these genes were higher in ovules, which was basically consistent with the RNA-seq data (Figure 9). The expression levels of *GH_A02G0519* and *GH_D02G0539* were higher in ovules, petals, and 30 DPA fibers. However, in the FLMN subgroup, the expression of *GH_A02G0216* in ovules was low, which was different from that in the database. The expressions of *GH_D02G0235*, *GH_A10G1391* and *GH_D10G1496* were basically consistent with those of the database, and all of them were highly expressed in the ovules, and *GH_D02G0235* was also highly expressed in the roots and sepals. According to the data from the fluorescence quantitative analysis and sequence analysis, the two genes closest to *At5g49150* were selected as candidate genes for subsequent research, in order to explore the influence of these two genes (*GH_A02G0519* and *GH_D02G0539*) on the reproductive development of upland cotton.

**Figure 10:**
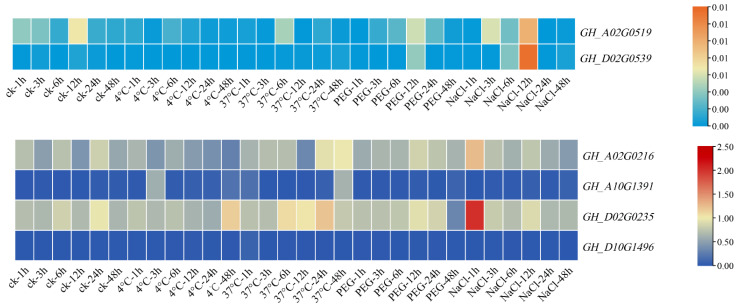
Heat map of *Filamin* gene family members in upland cotton expression under different abiotic stresses.

After the quantitative fluorescence analysis of these six genes in plants under different stresses, it was found that the results were basically consistent with the results from the RNA-seq database, and the gene expression showed a downward trend after abiotic stress (Figure 10). The downregulation range of *GH_A02G0519* and *GH_D02G0539* was not very large. The expression of *GH_A02G0519* decreased significantly when it was treated with a low temperature and high temperature for 12 h, but increased significantly when it was treated with NaCl for 12 h. The same was true for *GH_D02G0539*, which showed significant changes in expression after 12 h of treatment. However, the overall changes were small, and it can be inferred from the combined data in the database that these two genes are less sensitive to abiotic stress. The expression levels of *GH_A02G0216* and *GH_D02G0235* decreased significantly after low-temperature and PEG treatment, but the expression levels of *GH_A02G0216* and *GH_D02G0235* increased after 1 h of NaCl treatment and then decreased again, whereas the expression levels increased after high-temperature stress. The expressions of *GH_A10G1391* and *GH_D10G149* were downregulated under high-temperature, PEG and NaCl stress, and the expressions of *GH_A10G1391* and *GH_D10G149* were firstly decreased, then increased and, finally, decreased under low temperature. *GH_A02G0216* and *GH_D02G0235* showed great changes under stress. It is possible that these two genes have potential functions in abiotic stress, which need further study and verification.

### 3.10. Interaction Network of Filamin Proteins in Upland Cotton

**Figure 11:**
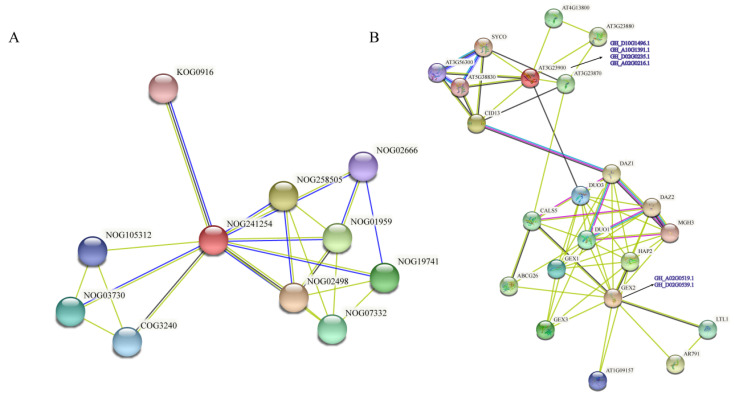
Interaction networks of filamin proteins. (**A**) Interaction network of filamin protein families. (**B**) Interaction network of filamin proteins in cotton with other proteins. The blue letters represent filamin proteins of upland cotton.

By predicting the protein interaction network of filamin proteins, their special function and relationship with other proteins can be understood deeply (Figure 11). A search of the entire protein family shows that the most central protein is filamin protein (NOG241254), and the closest interacting protein (NOG02498) plays a role in the single fertilization process, suggesting that filamin proteins may also play a role in the fertilization process. The filamin protein (NOG19741) also interacts with proteins in the organization of the inner membrane system. This protein belongs to the *DMP* family of membrane proteins, which has been shown to be associated with reproductive development and successful haploidy induction in cotton, suggesting that the filamin protein family also functions on the membrane [53]. For example, the GEX2 subfamily has been shown to be involved in gametophyte recognition and fusion in *A. thaliana*, and may have the same role in cotton reproductive development and even induce haploid production. The pathway of the 1, 3-β-glucan synthase/callose synthase catalytic subunit (KOG0916) has great significance in the reproductive development of angiosperms and in biotic and abiotic stresses [59]. In addition, another protein pathway (COG3240) belongs to phospholipase, which has also been proved to play a great role in cotton reproductive development. Through the role of these protein pathways, it can be speculated that filamin proteins may play a certain role in fertilization, gametophyte binding and other reproductive development processes.

A complete filamin protein network was obtained through a multisequence search of *Arabidopsis* filamin proteins. It is evident that there are many protein networks interacting with GEX2, and most of them are related to developmental fertilization. The closest pathway, *HAP2,* is expressed only in haploid spermatozoa, which are required for pollen tube guidance and fertilization [1,2,3,4,5], and are mainly localized in sperm endoplasmic reticulum membranes. *GEX1* [6,7,60], an interacting protein, has dual functions in gametophyte development and early embryogenesis, and thus, is required for proper pollen maturation. *GEX3*, a plasma membrane-localized protein expressed in male gametophytes, also plays a role in early embryogenesis. The interacting protein *DUO1* [61,62] activates germline-specific regulators that lead to pollen maturation and participate in pollen mitosis. *DAZ1* [63,64], *DAZ2* [63,64] and *MGH3* [65] proteins are closely related to the composition, development and fertilization of male gametophytes. This further proves that the GEX2 subfamily may be related to the development and growth of male gametophytes and fertilization. Among the interacting proteins in the FLMN subfamily, the *AT3G23880* pathway is a special one, as it contains the *F-box*-containing protein [66,67] and related interaction domains. Research shows that *F-box*-mediated target proteins were degraded in response to developmental and hormonal signals. Interestingly, this interacting protein *SYCO* [68] is required for female gametophyte development, unlike the GEX2 subfamily which is associated with male gametophytes, and may require further investigation.

## 4. Discussion

The *Filamin* gene family is mainly localized in cell membranes and has been reported to be involved in the composition of the human cytoskeleton, cancer cell metastasis and certain growth processes [14,15,16,17]. GEX2, a member of the *Filamin* gene family in plants, plays important roles in the processes of sperm recognition and fusion in *Arabidopsis* [3,20,21,22], *Zea mays* [24,25,26] and *Chlamydomonas* [27,28]. Due to the lack of research about *Filamin* gene family members in cotton, this paper conducted a systematic study on the cotton *Filamin* gene family and screened out genes that may have the same function as *Arabidopsis Filamin* gene family members, which was determined according to the structure, expression, evolutionary analysis and interaction analysis of these genes.

In this study, based on sequence analysis, phylogenetic tree construction analysis and gene structure analysis, filamin proteins can be divided into two subfamilies: GEX2 and FLMN. These two subfamilies have been found to be present in dicots and monocots, suggesting that the family was divided into two subgroups prior to the divergence of monocot and dicot crops. *Filamin* gene family members consist of exons and introns, and most *Filamin* gene family members contain 1–2 filamin domains, N-terminal signal peptides and C-terminal TM domains. Each different subgroup has essentially the same motif and domain, whereas the two motifs differ between the two subgroups, indicating that each subgroup is closely related to each other and that there are some differences between the subgroups. However, overall, evolution is conserved and the different motifs also show diversity in gene function. Among the 47 collinear pairs in the three cotton cultivars, most of them were whole-genome duplications (WGDs), which was the main reason for the evolution and expansion of the cotton genome, indirectly leading to its genetic diversity. Furthermore, Ka/Ks calculations for Ga-Gr and Gr-Gh collinearity pairs showed that only one collinearity pair had Ka/Ks > 1, whereas the others were all less than 1, suggesting that these genes have undergone purifying selections to eliminate deleterious mutations in the population during evolution, making the species more conserved and safe.

The interaction protein network analysis showed that cotton filamin proteins interact with many fertilization-related proteins, such as GEX1 [6,7,60], which plays a role in pollen maturation. GEX3 plays a role in early embryonic development; DAZ1 [63,64], DAZ2 [63,64] and MGH [65] are involved in the development, growth and fertilization of male gametophytes. These fully demonstrate the role of the GEX2 subfamily in reproductive development. The FLMN subfamily interacts with proteins containing F-box-related domains [66,67], and it is hypothesized that the *Filamin* gene family should also be involved in fertilization in cotton. At present, a point mutation in *At5g49150* belonging to the *Filamin* gene family in *Arabidopsis* has been found to affect the double fertilization of *Arabidopsis* due to the first base of the eighth intron [21,22].

By analyzing the *cis*-acting elements of the upstream promoter of the *Filamin* gene family member transcription start site in upland cotton, it was found that the promoter region contained many homogenic elements related to growth, development, abiotic stress and phytohormone signaling transduction.HD-ZIP1 is one of the special transcription factors [69] that plays a role in coping with abiotic stress and plant growth and development, and even causes plant growth retardation, senescence and death. According to the qRT-PCR analysis of these six genes, it can be seen that the two genes (*GH_A02G0519* and *GH_D02G0539*), which are closely related to *At5g49150* [21,22], are highly expressed in the ovule, and these two genes may also affect the double fertilization process in cotton. Secondly, the expressions of *GH_A02G0216* and *GH_D02G0235* tended to be significantly upregulated under high-temperature and NaCl stress, and these two genes could be used as candidate genes for studying abiotic stress. The GO analysis (Figure S5) of the *Filamin* gene family members in cotton as driver proteins also was mainly enriched in various ion transport pathways and indirectly affected its role in abiotic stress by regulating ion binding and tubulin [70,71]. Further functional verification is needed to determine whether these genes have a role.

## 5. Conclusions

Through a series of analyses of the sequences, gene structures, conservative domains, protein 3D structures, expression modes, *cis*-acting elements and interaction proteins of the *Filamin* gene family, it is shown that this family is mainly related to the reproductive development process. According to the fluorescence quantitative results and the analysis of the interaction protein, two genes related to reproductive development, *GH_A02G0519* and *GH_D02G0539*, are selected as the main functional genes for subsequent research. In this way, two genes (*GH_A02G0216* and *GH_D02G0235*) possibly related to stress were also found. This study preliminarily revealed the role of this gene family in cotton, providing a theoretical basis for the functional research of this family.

## Data Availability

The data presented in this study are available in the Supplementary Materials.

## Supplementary Materials

The following supporting information can be downloaded at: https://www.mdpi.com/article/10.3390/genes13122313/s1. Table S1: Basic information on 54 *Filamin* gene family members in 23 plant species. Table S2: Orthologous/paralogous gene pairs in three cotton species. Table S3: Analysis of *cis*-elements in upland cotton. Table S4: Expression values of *Filamin* gene family members of upland cotton in different tissues and different abiotic stresses obtained from RNA-seq data. Table S5: The primers used for qRT-PCR in this study. Figure S1: Statistics and evolution of species. Figure S2: Gene mapping of four cotton species. Figure S3: The synteny analysis of *Filamin* gene family members between *G. raimondii* and two representative plant species. The blue lines highlight the syntenic filamin gene pairs. The species names with the prefixes “Gh”, “Vv” and “Tc” indicate *G. raimondii*, *V. vinifera* and *T. cacao*, respectively. Figure S4: Conserved domains and motif analysis of *G. raimondii* and 19 species. Figure S5: GO enrichment analysis for cotton; black font represents GO number, and cyan font represents *p*-value.
