# Supplementary figures and images for: Evolutionary Relationships and Divergence of Filamin Gene Family Involved in Development and Stress in Cotton (Gossypium hirsutum L.)
Source: Genes (Basel). 2022 Dec 8;13(12):2313. doi: 10.3390/genes13122313 (PMC9777546; doi:10.3390/genes13122313)

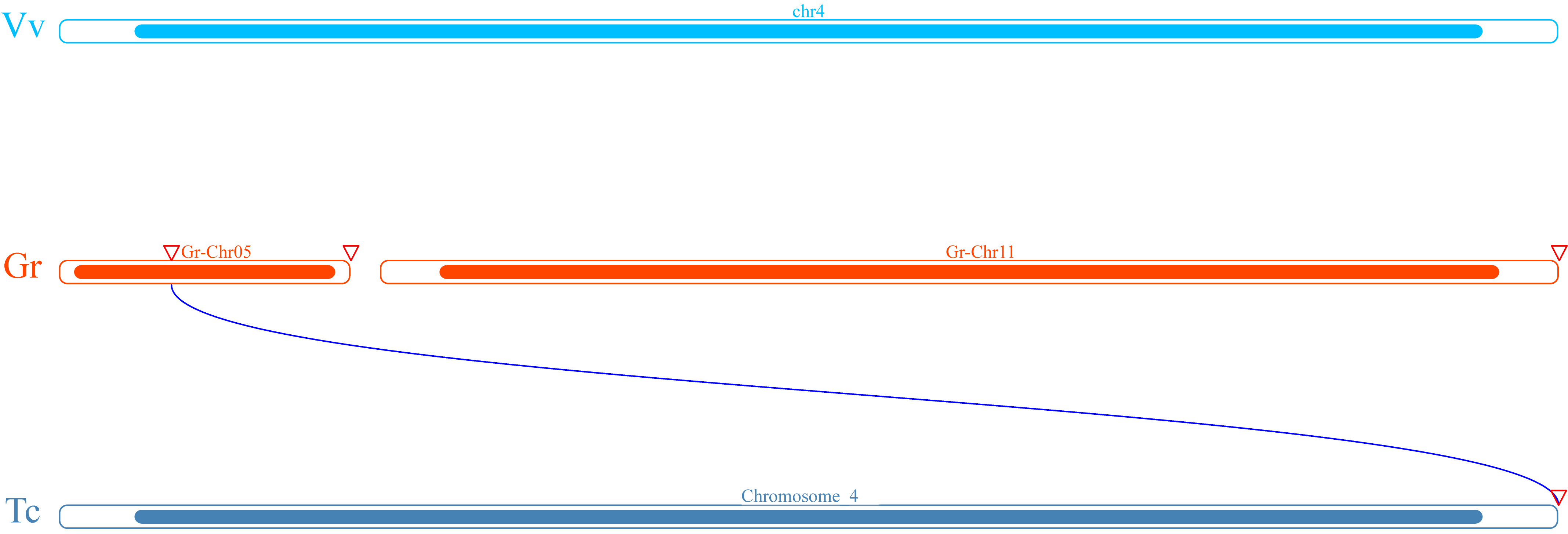

Supplement: Supplementary file 1 [file genes-13-02313-s001.zip › genes-2056137-supplementary/Supplementary Materials/Figure S3.tif]

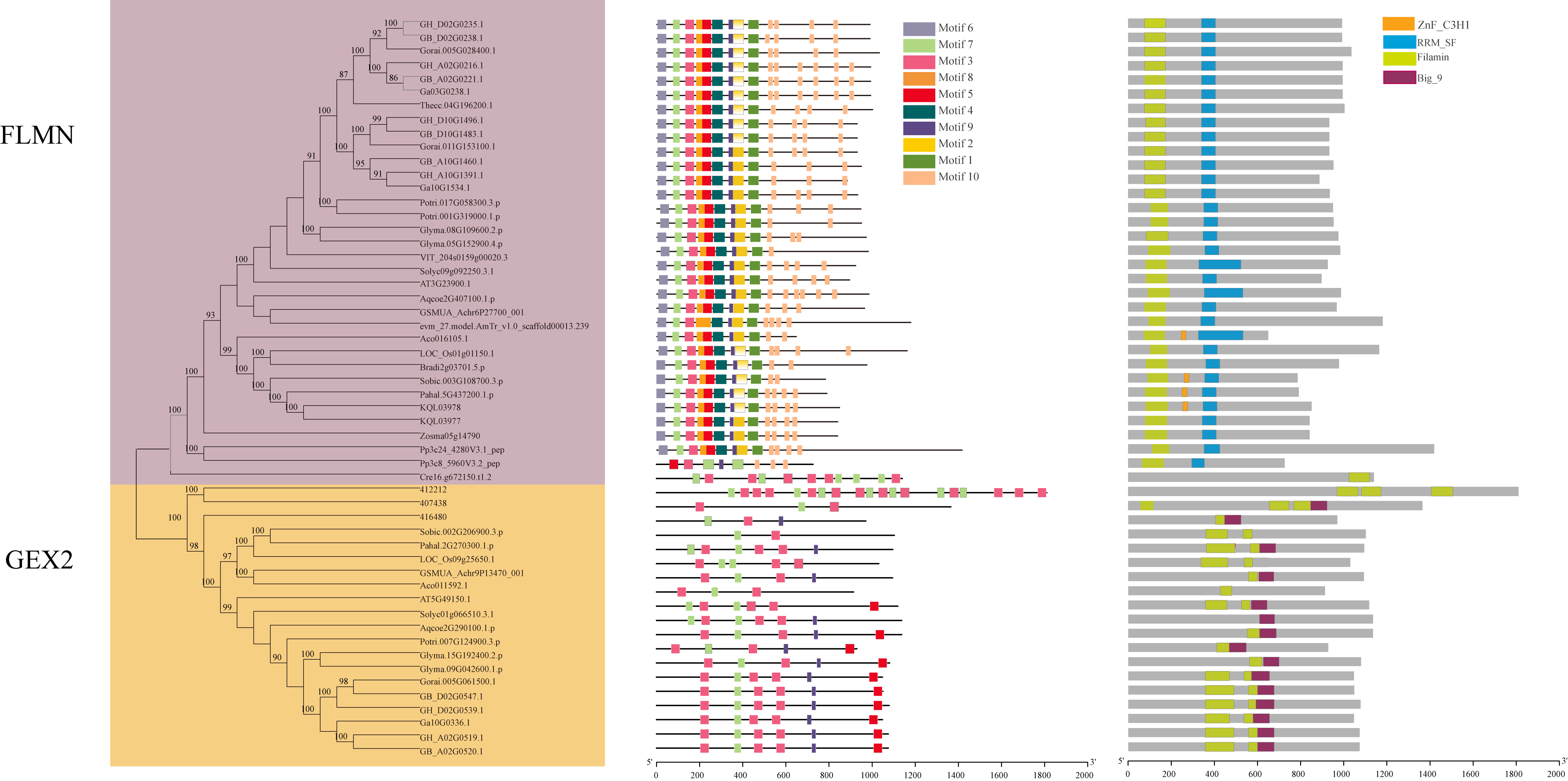

Supplement: Supplementary file 1 [file genes-13-02313-s001.zip › genes-2056137-supplementary/Supplementary Materials/Figure S4.tif]

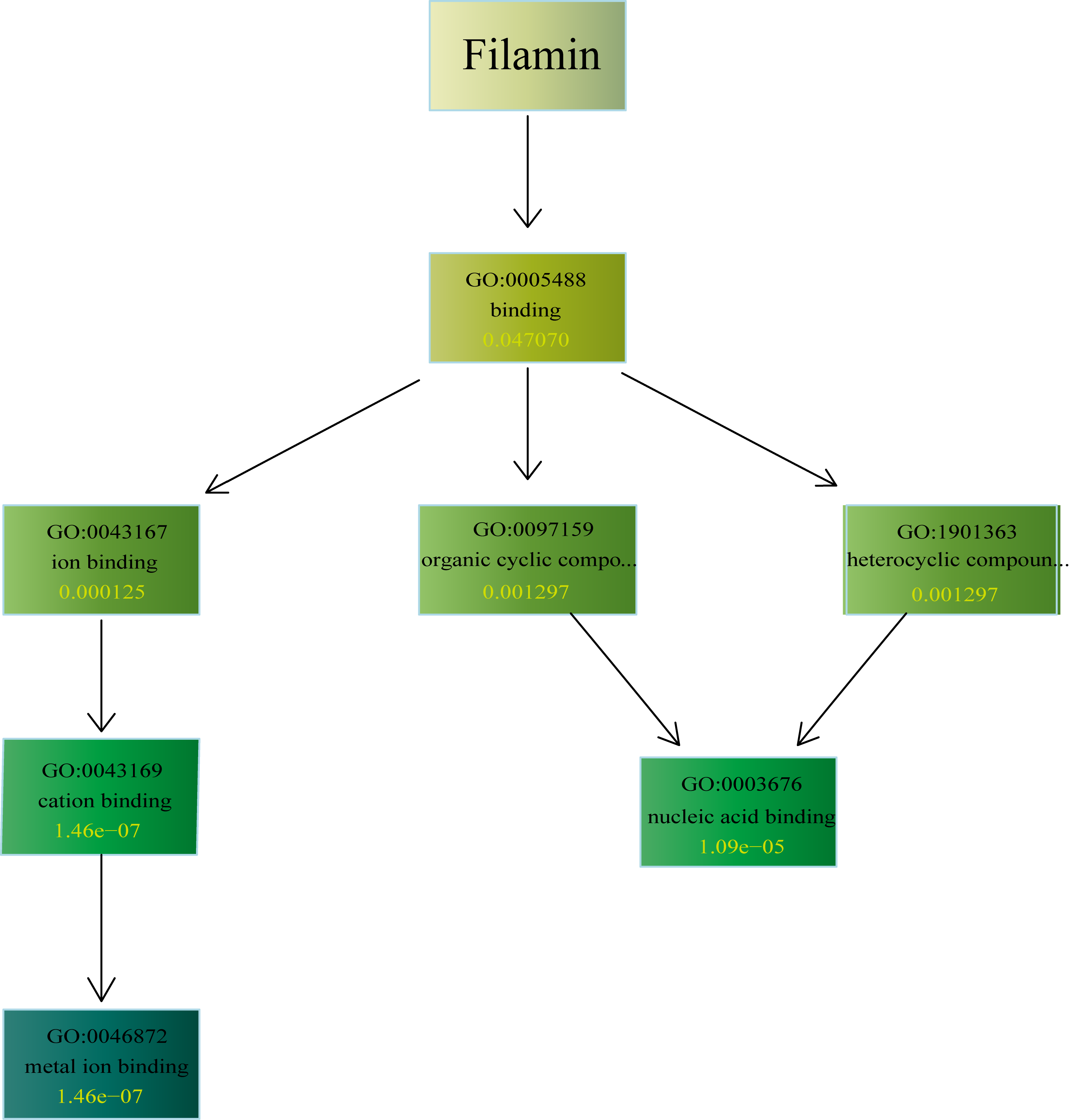

Supplement: Supplementary file 1 [file genes-13-02313-s001.zip › genes-2056137-supplementary/Supplementary Materials/Figure S5.tif]
